# Evaluating the Effectiveness of Digital Interventions for Stress Management in Pregnant Women: Systematic Review and Meta-Analysis

**DOI:** 10.2196/66267

**Published:** 2026-01-26

**Authors:** Jeung-Im Kim, Joo Yun Lee, So-Hee Park

**Affiliations:** 1 School of Nursing, College of Medicine, Soonchunhynag University Asan-si Republic of Korea; 2 College of Nursing, Gachon University Incheon Republic of Korea; 3 School of Nursing, College of Medicine, Soonchunhyang University Asan-si Republic of Korea

**Keywords:** pregnancy, stress management, digital health, mHealth, telemedicine, systematic review, meta-analysis

## Abstract

**Background:**

Psychological stress during pregnancy is common and has been associated with adverse maternal and neonatal outcomes. Digital health interventions (DHIs) have emerged as a scalable approach to support stress management during pregnancy, yet evidence remains fragmented, and prior reviews have largely focused on broad perinatal mental health outcomes or delivery platforms rather than stress-specific effects and targeted intervention components.

**Objective:**

This systematic review and meta-analysis aimed to evaluate the effectiveness of DHIs specifically designed to reduce stress during pregnancy and to examine how intervention strategies and delivery methods are associated with stress outcomes.

**Methods:**

We conducted a systematic review and meta-analysis following PRISMA (Preferred Reporting Items for Systematic Reviews and Meta-Analyses) 2020 guidelines. Randomized controlled trials and quasi-experimental studies involving pregnant women were eligible if they evaluated any digitally delivered intervention—such as mobile apps, web-based programs, or telemedicine—intended to reduce stress, and reported validated stress outcomes. We searched CINAHL, the Cochrane Library, Embase, and PubMed from database inception through November 2025. Risk of bias was assessed using the Cochrane risk of bias 2 tool for randomized trials and the risk of bias in nonrandomized studies of interventions tool for nonrandomized studies. Where appropriate, effect sizes were pooled using random-effects meta-analysis with the Hartung–Knapp–Sidik–Jonkman method and reported as standardized mean differences.

**Results:**

A total of 19 studies were included. Overall, DHIs were associated with a significant reduction in stress compared with control conditions (standardized mean difference –0.45, 95% CI –0.59 to –0.32; 95% prediction interval –0.78 to –0.13), with low to moderate heterogeneity. Strategy-based subgroup analyses indicated that mindfulness- and education-focused interventions showed favorable effects, but formal tests for between-subgroup differences were not statistically significant. Evidence certainty was rated as moderate, primarily due to risk-of-bias concerns in some trials.

**Conclusions:**

This review provides stress-focused evidence that DHIs can support stress reduction during pregnancy and extends existing literature by systematically disaggregating interventions according to delivery methods, functional features, and content strategies. This study offers a component-oriented synthesis that informs the design and selection of digital stress-management interventions for pregnant women. In real-world antenatal care, these tools may complement clinician-delivered services by expanding access to low-intensity, scalable support, particularly when interventions integrate skills-based content with supportive digital functions. Future research should directly compare single versus combined strategies and evaluate implementation across diverse populations and care settings.

## Introduction

### Overview

Pregnancy represents a major life transition that is frequently accompanied by heightened psychological stress, including pregnancy-specific stress related to concerns about maternal health, fetal well-being, and childbirth. Recent evidence indicates that pregnancy-specific stress is prevalent even among low-risk populations and tends to increase with perceived pregnancy risk and advancing gestational age [1]. Elevated stress during pregnancy has been consistently associated with adverse maternal mental health outcomes as well as negative neonatal and child health outcomes, underscoring the importance of timely and effective stress management during the antenatal period [2-4].

A range of nonpharmacological interventions has been developed to address stress during pregnancy, and their effectiveness has been demonstrated across multiple studies. These include psychoeducational programs, structured psychotherapies such as cognitive behavioral therapy and interpersonal psychotherapy, and mind–body approaches including mindfulness and yoga [5-8]. Traditionally, such interventions have been delivered through face-to-face sessions. While effective, in-person delivery can be constrained by barriers related to access, cost, time, geographic distance, and the availability of trained professionals—limitations that may be particularly salient during pregnancy.

Digital health interventions (DHIs) have emerged as a scalable and low-threshold approach for delivering stress-management strategies, with growing evidence supporting their effectiveness in the general population. Meta-analytic evidence indicates that app-based stress-management interventions yield small but statistically significant improvements across self-reported, physiological, and neuroendocrine stress outcomes [9], and similar modest reductions in perceived stress have been reported in randomized trials of mental health smartphone apps [10]. These findings suggest that digitally delivered interventions can effectively support stress reduction when designed and implemented appropriately.

However, evidence specific to pregnancy remains more limited and less conclusive. Much of the existing literature on DHIs during pregnancy has focused on broader perinatal mental health outcomes—particularly depression and anxiety—rather than stress as a primary target. For example, reviews of digital mindfulness and nurse-led eHealth interventions have reported consistent benefits for depressive and anxiety symptoms but mixed or inconclusive effects on stress [11,12], while telemedicine-based psychological interventions often combine antenatal and postpartum populations and rarely prioritize stress outcomes [13]. An umbrella review of DHIs for perinatal women suggested overall benefits for psychological outcomes, including stress; however, its findings were pooled across heterogeneous populations, intervention types, outcomes (stress, depression, and anxiety), and perinatal stages, with only a small subset of reviews specifically addressing stress during pregnancy [14]. Collectively, these syntheses highlight that stress-focused evidence in pregnant populations remains fragmented and underdeveloped.

At the same time, DHIs offer distinct methodological and practical advantages that warrant more nuanced evaluation. Digital interventions are inherently multicomponent, comprising combinations of delivery methods (eg, mobile apps, web-based platforms, and telemedicine), functional features (eg, self-monitoring, automated feedback, and reminders), and intervention contents (eg, psychoeducation, mindfulness, relaxation, and cognitive behavioral skills). Behavioral science frameworks emphasize that technology functions primarily as a delivery vehicle rather than the therapeutic agent itself [15], and empirical assessments of stress-management apps similarly distinguish delivery modalities, functional features, and therapeutic content as separable but interacting components [16]. Despite this, prior reviews of digital mental health interventions during pregnancy have rarely synthesized evidence across these dimensions, limiting understanding of which combinations of delivery methods, functions, and contents are most relevant for stress reduction in pregnant women.

### Rationale

Taken together, the existing literature highlights a persistent gap in evidence regarding digital interventions specifically designed to reduce stress during pregnancy. Although DHIs for perinatal mental health have received growing attention, most prior reviews have focused primarily on depression and anxiety or have pooled multiple psychological outcomes, with stress often insufficiently examined as a primary outcome. As a result, the effectiveness of digital interventions targeting stress during pregnancy remains unclear.

Moreover, previous syntheses have largely categorized digital interventions by delivery platform, providing limited insight into how specific intervention components contribute to stress reduction. DHIs are inherently multicomponent, integrating delivery methods, functional features, and therapeutic content; however, the relative importance of these components for managing pregnancy-related stress has not been systematically examined.

To address these gaps, the present systematic review and meta-analysis provide an updated and focused synthesis of digital stress-management interventions during pregnancy. By centering stress reduction as the primary outcome and examining intervention effectiveness through the lens of delivery methods and intervention content, this review aims to clarify how digital interventions can be optimally designed and implemented to support stress management in real-world antenatal care settings.

### Objectives

Following PRISMA (Preferred Reporting Items for Systematic Reviews and Meta-Analyses) 2020 guidance [17] and PRISMA-S [18], this systematic review aimed to (1) identify and describe digital stress-management interventions for pregnant women (Population), including delivery platforms and key components (Intervention); (2) evaluate the effectiveness of these interventions compared with usual care, wait-list, or active comparators (Comparator) on validated stress outcomes (Outcome); and (3) summarize intervention strategies and delivery methods associated with engagement and stress reduction.

## Methods

### Overview

We conducted a systematic review and meta-analysis to evaluate digitally delivered interventions for reducing stress in pregnant women. The review adhered to the PRISMA guidelines [17] and PRISMA-S (PRISMA literature search extension) [18] (Multimedia Appendix 1).

### Eligibility Criteria

Eligibility criteria were predefined using the PICOS (population, intervention, comparison, outcome, and study design) framework. Participants were pregnant women. Interventions included any digitally delivered program primarily aimed at reducing or managing stress during pregnancy (antenatal period), delivered via mobile apps, web-based platforms, telemedicine, or other technology-enabled modalities. Studies in which the intervention was initiated postpartum were excluded. Comparators included usual care, waitlist/no intervention, or nondigital interventions. Outcomes included stress assessed using validated instruments; when the Depression Anxiety Stress Scales (DASS) was used, only the stress subscale was extracted. Study designs included RCTs and quasi-experimental/nonrandomized intervention studies with extractable pre-post outcome data.

Exclusion criteria included (1) postpartum-only interventions (interventions initiated after delivery), (2) interventions not primarily targeting stress management (eg, programs targeting depression, weight control, smoking cessation, or other conditions in which stress was only a secondary outcome without a stress-focused rationale), (3) insufficient outcome data to estimate effects (no extractable pre-post data or between-group comparisons), and (4) conference abstracts or trial registry entries without sufficient methodological detail or outcome data for extraction and risk-of-bias assessment.

### Information Sources

The initial systematic search was conducted across 4 databases (CINAHL, Cochrane Library, Embase, and PubMed) from database inception to September 2023. In response to reviewer comments and to ensure the currency of the evidence base, the search was updated by re-running the same search strategy in all databases for records published from September 2023 through November 30, 2025. The updated search was executed on December 10, 2025, by JYL and SHP, and all newly retrieved records were screened and incorporated into the final synthesis as appropriate. Searches were conducted with the assistance of 2 medical librarians, and the final search strategies for each database are reported in Multimedia Appendix 2. Each database was searched independently using its native platform (PubMed, Embase.com, CINAHL via EBSCOhost, and the Cochrane Library). Databases were not searched simultaneously on a single platform. In addition to database searching, reference lists of included studies were manually screened to identify potentially relevant articles using citation searching methods. We did not contact study authors, experts, or intervention developers to obtain additional data, nor were other information sources such as trial registries or gray literature repositories searched.

### Search Strategy

Electronic search strategies were developed in consultation with 2 medical librarians, who proposed candidate keywords and controlled vocabulary; the research team reviewed and refined the strategies, and final searches were executed by the study team with librarian support. All search strategies were newly developed for this review and were not adapted from or reused based on search strategies reported in previous literature reviews. The strategy combined controlled vocabulary (MeSH [Medical Subject Headings], Emtree, and CINAHL Subject Headings) and keywords related to pregnancy, digital health, and stress. Pregnancy-related terms included “pregnan*,” “pregnancy,” “antenatal,” and “prenatal” (and related indexing terms). Digital health terms included “digital health,” “mHealth,” “eHealth,” “telemedicine,” “mobile app,” and “smartphone.” Stress-related terms included “stress” and relevant intervention terms (eg, relaxation, mindfulness, cognitive behavioral). Animal studies were excluded, and results were limited to English-language publications. Full search strategies for each database are provided in Multimedia Appendix 2.

### Selection Process

All records were imported into EndNote and deduplicated. Three reviewers (JIK, JYL, and SHP) independently screened titles and abstracts to identify potentially eligible studies. Full texts were retrieved for records deemed relevant or uncertain; when full-text articles were difficult to obtain, a medical librarian assisted with document retrieval. Two reviewers (JIK and SHP) independently assessed full-text eligibility and cross-checked decisions, with disagreements resolved through discussion (and adjudication by a third reviewer when necessary). For the updated search, a second screening pass was completed on December 12, 2025, and all newly retrieved articles were screened and assessed for eligibility using the same procedures as the initial review, with eligible studies incorporated into the final synthesis.

### Data Collection Process and Data Items

Data was extracted using a piloted structured form capturing intervention purpose, participant characteristics, timing and duration, intervention strategies/components and digital functions, delivery mode, comparator, sample size, stress measures, and outcome values. When outcomes were measured repeatedly, the assessment closest to intervention completion was extracted; follow-up outcomes were extracted separately where available. Two reviewers independently entered data into an Excel file and verified consistency. Discrepancies were resolved by consensus among 3 reviewers to ensure accuracy and consistent terminology.

### Study Risk of Bias Assessment

Risk of bias was assessed using RoB 2 (a revised Cochrane risk-of-bias tool for randomized trials) for randomized trials [19] and ROBINS-I (risk of bias in nonrandomized studies of interventions) for quasi-experimental/nonrandomized studies [20]. Two reviewers (JIK and SHP) independently rated each study, and disagreements were resolved through discussion with adjudication by a third reviewer [JYL] when needed. RoB 2 judgments were made across 5 domains and summarized as low risk, some concerns, or high risk. ROBINS-I judgments were made across 7 domains and summarized as low, moderate, serious, or critical risk of bias. Risk-of-bias assessments informed sensitivity analyses and the certainty of evidence (GRADE [Grading of Recommendations, Assessment, Development and Evaluation]).

### Effect Measures

The primary outcome was the change in stress from baseline to postintervention (closest to intervention completion). When DASS was used, only the stress subscale was extracted. Continuous outcomes were synthesized as standardized mean differences (SMDs; Hedges *g*) and reported with 95% CIs [21], with negative values indicating lower stress in the intervention group. When studies did not report means and SDs directly, effect estimates were derived from available statistics (eg, *F* values, odds ratios, chi-square values) using standard conversion methods.

### Synthesis Methods

Study characteristics (population, intervention components and digital functions, delivery mode, stress measures, and effects) were summarized narratively. For quantitative synthesis, meta-analyses were conducted using random-effects models to reflect expected between-study variability in true effects. Analyses were performed in in R (version 4.5.2; R Foundation for Statistical Computing, Vienna, Austria) using the Hartung–Knapp–Sidik–Jonkman method [22]. Heterogeneity was quantified using τ² and *I*², with the Cochran Q test reported where appropriate. To distinguish the average pooled effect from the expected distribution of effects across settings, we reported 95% prediction intervals for the main meta-analyses (but not for subgroup analyses). CIs describe uncertainty around the pooled mean effect, whereas prediction intervals indicate the plausible range of effects in a new setting. Prespecified subgroup analyses (eg, intervention strategies/components and delivery modes) and, when sufficient studies were available, meta-regression were used to explore potential sources of heterogeneity. Sensitivity analyses excluded studies at high or serious risk of bias and tested alternative assumptions for imputed parameters where applicable. Small-study effects were assessed using funnel plots and Egger’s regression test when ≥10 studies were available for a given main analysis.

### Certainty Assessment

Two reviewers (JIK and SHP) independently assessed certainty of evidence using GRADE, with disagreements resolved through consensus and third-reviewer adjudication. Certainty was evaluated across risk of bias, inconsistency, indirectness, imprecision, and publication bias. Downgrading decisions were documented in the Summary of Findings table and were informed by RoB 2/ROBINS-I judgments, heterogeneity (including the extent to which prediction intervals indicated variability across settings), imprecision (width of CIs and information size), and evidence of small-study effects (funnel/Egger).

## Results

### Study Selection

**Figure 1:**
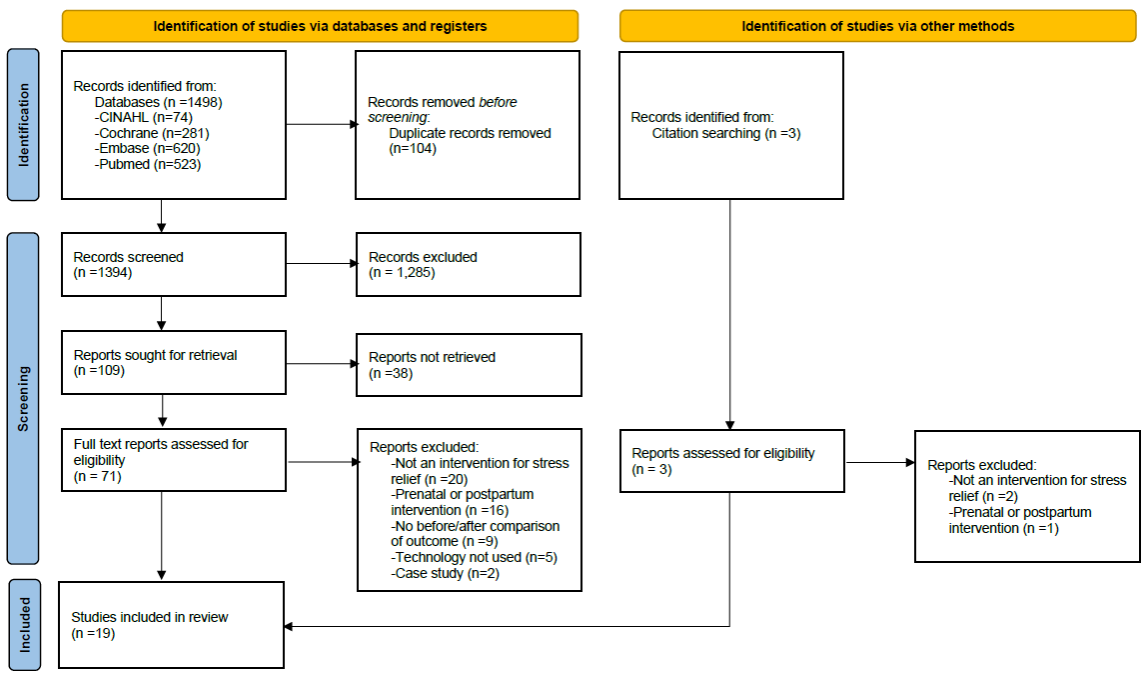
Study selection process based on the PRISMA (Preferred Reporting Items for Systematic Reviews and Meta-Analyses) 2020 flow diagram.

The study selection process is summarized in Figure 1. Studies were excluded if they targeted health conditions other than stress (eg, depression or anxiety, weight control, or smoking cessation) without a stress-focused intervention rationale, or if stress was measured only as a secondary outcome. Studies describing intervention development without reporting evaluable effectiveness outcomes were also excluded. Citation searching identified additional records, which were assessed at the full-text level but ultimately did not meet the inclusion criteria. Reasons for exclusion at each stage were documented and are presented in the PRISMA flow diagram.

Following the updated search and eligibility assessment of newly retrieved records, 4 additional studies were included. In total, 19 studies (15 from the initial search and 4 from the updated search) were included in the final review.

### Study Characteristics

Included studies were published between 2016 and 2025 [23-41]. Among them, 7 studies [24,26,28,31,33,34,38] were conducted in the United States (37%), followed by studies from Iran (n=2) [29,37], China (n=2) [25,34], Korea (n=2) [24,32], and other countries. Overall, there were 12 RCTs (63%) and 7 quasi-experimental studies (37%; Table 1).

**Table 1:** Characteristics of the included studies.

Author			Country (year)		Target	Gestational age at intervention		Intervention period		Delivery mode		Intervention contents		Intervention details
**Randomized controlled study**														
	Balderas-Díaz et al [23]	Spain (2022)		At risk of having SGA^a^ fetuses		Second trimester and part of the third trimester	16 weeks		Mobile		Education and information		Learn about specific topics, carry out the proposed activities to strengthen training Four components: Medical advice, health care, communication with the fetus for stimulation purposes, and emotional management	
	Lee et al [24]	Korea (2023)		Working pregnant women		From ≤34 weeks	4 weeks		Mobile		Education and information with peer support		Four components: Eight education sessions, a health log, a diary, and an anonymous discussion board Each session lasts approximately 15 minutes Four targeted practices: Sleep and rest, eating, physical activity, and stress management	
	Sun et al [25]	China (2021)		At risk of perinatal depression (with an EPDS^b^ score >9 or a PHQ-9^c^ score >4)		12-20 weeks	8 weeks		Mobile		Mindfulness		Formal mindfulness training: Body scan, mindful breathing, mindful stretching, and mindful meditation lasting 15-25 minutes per day Informal training: Pausing in the midst of daily life, mindful eating, mindful walking, and 3-minute breathing practices	
	Smith et al [26]	United States (2021)		Obstetric patients of outpatient clinic during the COVID-19 pandemic		14-34 weeks	30 days		Mobile		Mindfulness		Mindfulness meditation, sleep stories, and nature sounds Encourage use of 70 minutes per week, preferably 10 minutes per day	
	Krusche et al [27]	United Kingdom (2018)		Pregnant women recruited online		12-34 weeks	4 weeks		Website		Mindfulness		Formal and informal meditation practices such as body scan, mindful movement, breathing space, and mindful eating Online sessions, assignments, and emails	
	Dennis-Tiwary et al [28]	United States (2017)		Pregnant women recruited from a large urban hospital		19-29 weeks	4 weeks		Mobile		Game		Gamified attention bias modification training (ABMT), incorporating video game-like features such as animated characters and sound effects 10 rounds each day (≤10 minutes of play) for 4 days/week: 160 rounds total for the duration of the study	
	Kia [29]	Iran (2023)		No stressful event other than COVID-19 disease in the preceding 6 months		12 or more weeks	3 weeks		Mobile		Education and information		Educational theme: (1) COVID-19 disease explanation, personal hygiene, and transmission modes, (2) COVID-19 in pregnancy and childbirth, (3) COVID-19 and breastfeeding, (4) COVID-19 and infants Five sessions of 30 minutes, 2 sessions per week	
	Mauriello et al [30]	United States (2016)		Pregnant women recruited from 6 locations of 3 federally funded health centers		From <19 weeks	24 weeks		Mobile		Education and information		Stage-matched and tailored guidance, 3 interactive sessions focused on 2 priority health behavior risks (smoking, stress management, fruits and vegetables), individually tailored and stage-matched change strategies Feedback messages within stage-matched activities, including tools such as calculators, quizzes, action plans, support messages, and recipe ideas Three antenatal sessions are spaced approximately 12 weeks apart, with 2 postpartum assessment-only sessions at 1 month and 4 months	
	Chua et al [31]	Singapore (2024)		Heterosexual couples recruited from a public tertiary hospital		From >24 weeks	From 24 weeks to 1 month postpartum		Mobile		Education and information and support		Six components: (1) Education center (multimedia resources, chatbot), (2) Ask an expert (Experienced nurses/midwives will respond to queries within 24 h), (3) Smile center (mood rating, guided mindfulness), (4) Positivity space (support forum), (5) Gamification features (virtual badges, rewards), (6) Helpline	
	Park et al [32]	Korea (2025)		Pregnant women recruited from an obstetrics and gynecology center		1-32 weeks	8 weeks		Mobile		Mindfulness		Four sections: (1) breathing mindfulness meditation, (2) body scanning, (3) emotional awareness, (4) self-kindness mindfulness Each session was divided into 2 subsessions—instruction and practice sessions. Instructed to practice each session at least twice	
	Tandon et al [33]	United States (2025)		Pregnant women recruited from 6 university affiliated prenatal care clinics		From <22 weeks	maximum of 14 weeks		Mobile, in-person		Education and Mindfulness		Twelve-session manualized intervention: CBT^d^ content related to behavioral activation, identification, and reframing unhelpful thought patterns, and promotion of positive interactions Includes various mindfulness practices Received just-in-time (JIT) messages were sent within 24 h of an elevated stress reading	
	Tian et al [34]	China (2025)		Couples expecting their first child recruited from outpatient centers		12-20 weeks	6 weeks		Mobile		Mindfulness		Six one-week modules, each with a thematic session and 6 audio-guided home practice sessions Mindfulness exercises that involved couple collaboration and interaction	
**Quasi-experimental study**														
	Porter et al [35]	United States (2022)		Pregnant women recruited from a university hospital		<15 weeks to 28 weeks	13 weeks		Mobile		Mindfulness		Meditations tailored to the trimester and specific physical and emotional states of participants Recommend daily usage of 10-20 minutes	
	Jallo et al [36]	United States (2017)		Hospitalized with preterm labor		22+0-33+6 weeks	8 days		Mobile		Education and relaxation		Educational overview (stress, stress response, and impact on health) Four guided imagery audio files, including relaxation, focused breathing, positive affirmations, and multiple multisensory images, each lasting 15-20 minutes long A stress self-assessment scale	
	Hashemzahi et al [37]	Iran (2022)		With perceived stress (PSS^e^ score ≥21.8) and moderate to severe anxiety (CDAS^f^ score ≥17)		20-28 weeks	2 weeks		Telemedicine (WhatsApp)		Education and support		Educational content covered topics such as familiarity with COVID-19, its effect on pregnancy, prevention and self-care during pregnancy, and guidelines for care and prevention during childbirth, postpartum, and breastfeeding The content was shared with the group every other day over 2 weeks, comprising 6 sessions via WhatsApp. The researcher followed up to ensure that participants watched the educational videos and addressed any questions or concerns raised by participants	
	Tsai et al [38]	Taiwan (2018)		Low-risk pregnant women in the outpatient department of a medical center		From 16-24 to 36-38 weeks	12-22 weeks		Website (accessible via smartphone and computer)		Education and information		Four modules: Maternity health records, antenatal health education, self-management journals, newborn birth records Participants could store their own information and records Automatic pop-up windows provide antenatal health information according to a woman’s gestational age	
	Buultjens et al [39]	Australia (2023)		Low-risk pregnant women at one community health site		From 28-30 weeks to 36-38 weeks	6-10 weeks (Antenatal)		Telemedicine (video conference)		Education and support		One-to-one pregnancy clinical care, integrating standard pregnancy health assessments with structured online small-group interdisciplinary education and peer support, thus incorporating broader psychosocial aspects Four group antenatal and 4 group postnatal education sessions	
	Kubo et al [40]	United States (2021)		With moderate-to-moderately-severe depressive symptoms (PHQ-8^g^ score 10-19)		From <28 weeks	6 weeks		Mobile		Mindfulness		Basic mindfulness condition- or situation-specific courses Headspace courses including breathing exercises, body scan, noting, and visualization Additional short (1-2 min) lecture videos designed to increase the understanding of mindfulness and encourage its integration into daily life Participants asked to use the app for 10-20 min a day over 6 weeks	
	Barber and Masters-Awatere [41]	New Zealand (2022)		Pregnant women recruited from midwifery clinics, through antenatal educators, and via social media		<24 weeks	12 weeks		Mobile		Education and relaxation		Four types of modules: (1) interactive self-assessments with associated feedback, (2) activities for relaxation, stress management and planning for parenthood, (3) information about psychological and social changes in pregnancy, and (4) discussions with a partner or support person to build social support and interactive reflection and problem-solving	

^a^SGA: small for gestational age.

^b^EPDS: Edinburgh Postnatal Depression Scale.

^c^PHQ: patient health questionnaire.

^d^CBT: cognitive behavioral therapy.

^e^PSS: Perceived Stress Scale.

^f^CDAS: Corona Disease Anxiety Scale.

^g^PHQ-8: Patient Health Questionnaire-8.

### Risk of Bias in Studies

**Figure 2:**
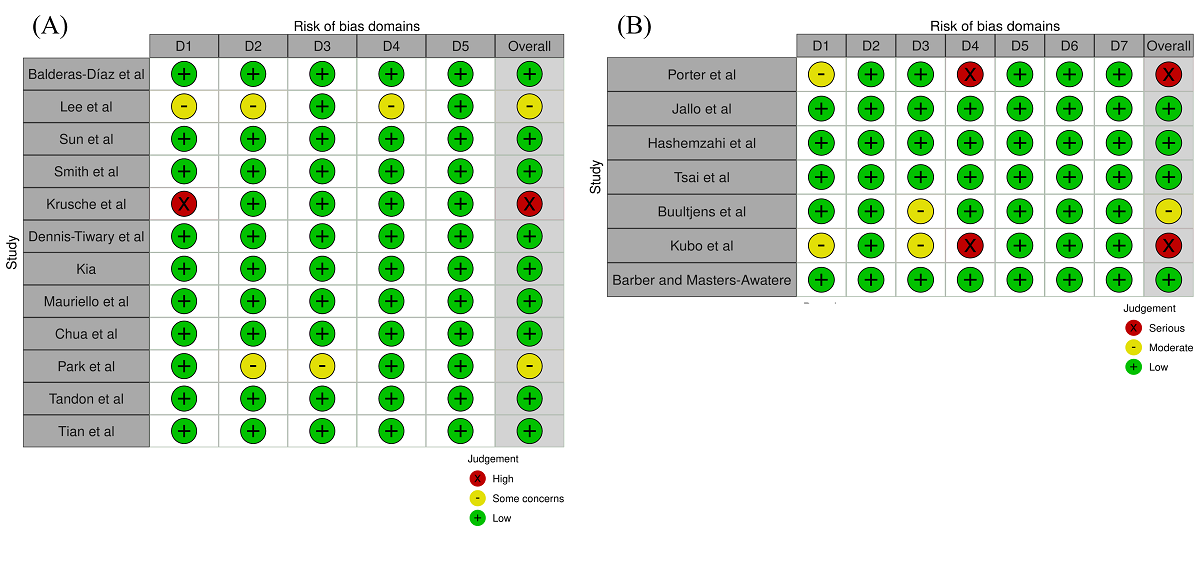
Risk of bias assessments. (A) Risk of bias for randomized controlled trials assessed using the revised Cochrane risk-of-bias tool. Domains: D1 (randomization process), D2 (deviations from intended interventions), D3 (missing outcome data), D4 (measurement of the outcome), and D5 (selection of the reported result). (B) Risk of bias for nonrandomized studies assessed using the risk of bias in nonrandomized studies of interventions. Domains: D1 (confounding), D2 (selection of participants), D3 (classification of interventions), D4 (deviations from intended interventions), D5 (missing data), D6 (measurement of outcomes), and D7 (selection of the reported result) [23-41].

The results of the risk of bias assessment are presented in Figure 2 [23-41]. Among the 12 RCTs assessed with RoB 2, one study was judged at high risk of bias overall, driven by concerns in the randomization process (Domain 1). Two additional RCTs were rated as having some concerns overall, reflecting incomplete reporting or concerns in specific domains, while the remaining RCTs were judged at low risk of bias across domains.

Among the 7 nonrandomized studies assessed with ROBINS-I, 2 studies were judged at serious risk of bias overall, primarily due to deviations from intended interventions (Domain 4). One study was judged at moderate risk of bias, and the remaining studies were judged at low risk across ROBINS-I domains. No study was judged at critical risk of bias.

### Results of Individual Studies

#### Study Population

All studies targeted pregnant women, with 12 studies focusing on low-risk pregnancies (63%), while the remaining studies targeted pregnant women with specific issues or risk factors. Two studies focused on women at risk of perinatal depression, while one study each targeted women with perceived stress, stress induced by the COVID-19 pandemic, small for gestational age fetuses, and those hospitalized due to preterm labor. Additionally, one study focused on stress management in working pregnant women. Among the 12 studies, 9 studies in the low-risk group primarily started in the second trimester and often continued into the third trimester. Interventions targeting pregnant women with depression or stress and those carrying small for gestational age fetuses started in the second trimester. Interventions for preterm labor were initiated between 22 and 33 weeks of gestation. Meanwhile, interventions for working pregnant women could be initiated at any time before 34 weeks of gestation.

#### Intervention Strategies

When categorizing the intervention strategies, 4 studies provided only education and information (21%), while 7 studies incorporated additional strategies (37%). Specifically, 2 studies included professional support, and 2 studies incorporated peer support, which we classified as the strategy of “seeking support.” Three studies included relaxation content, such as guided imagery audio files or activities for relaxation, such as watching humorous YouTube videos. One of these studies also included peer support. Furthermore, 7 studies primarily focused on mindfulness, and one study offered an intervention in the form of a game (Table 2).

**Table 2:** Intervention strategies and functions included in the stress management intervention.

Category and details				Study
**Intervention strategies**				
	**Education and information**			
		Education, medical advice: text, video files, automatic pop-up, or videoconference	Barber and Masters-Awatere [41], Balderas-Díaz et al [23], Lee et al [24], Kia [29], Jallo et al [36], Hashemzahi et al [37], Tsai et al [38], Buultjens et al [39], Chua et al [31], Tandon et al [33]	
	**Seeking support**			
		Anonymous discussion board	Lee et al [24], Chua et al [31]	
		Online group peer support	Buultjens et al [39]	
		Conversation with the partner, other supporters	Barber and Masters-Awatere [41]	
		Question and answer from the researcher	Hashemzahi et al [37]	
	**Relaxation**			
		Guided imagery audio files	Barber and Masters-Awatere [41], Jallo et al [36]	
		Activities for relaxation	Barber and Masters-Awatere [41]	
	**Mindfulness**			
		Formal training: body scan, mindful breathing, mindful stretching, mindful meditation, and visualization: text, video files, and audio files	Sun et al [25], Smith et al [26], Krusche et al [27], Porter et al [35], Kubo et al [40], Park et al [32], Tandon et al [33], Tian et al [34]	
		Informal training: mindful eating, mindful walking	Sun et al [25], Krusche et al [27]	
	**Game**			
		Attention bias modification training	Dennis-Tiwary et al [28]	
**Functions**				
	**Monitoring (recording)**			
		Health log, diary	Lee et al [24], Tsai et al [38], Tandon et al [33]	
		Mindfulness journal	Sun et al [25]	
		Self-assessment scale	Barber and Masters-Awatere [41], Jallo et al [36]	
	**Automated feedback**			
		Tailored guidance, feedback messages: programmed messages and chatbot	Barber and Masters-Awatere [41], Mauriello et al [30], Chua et al [31], Tandon et al [33]	
	**Reminder**			
		Notifications and reminders to prompt use	Barber and Masters-Awatere [41], Sun (WeChat) et al [25], Krusche (e-mail) et al [27], Kubo et al [40], Tian et al [34]	

In the process of content extraction, some elements were classified as functions according to the categorization by Paganini et al [16]. For instance, monitoring (recording) included studies where participants regularly assessed stress or fetal development through health logs, diaries, or self-assessment tools. Automated feedback involved the delivery of tailored guidance and feedback messages through programmed communication. There was an intervention incorporated with interactive chatbot functions that responded to users’ questions in real time, providing guidance related to stress management and pregnancy. Additionally, 4 studies included a reminder function, which prompted participants to re-engage with the digital intervention if they had not participated for a certain period.

#### Intervention Delivery Mode

The digital interventions, which were the focus of the studies, were delivered via mobile app in 15 (79%) studies, via the web in 2 (10.5%) studies, and via telemedicine in 2 studies, using video conferencing and WhatsApp Messenger.

#### Measure of Stress

Stress was measured using various tools. Ten (53%) studies used the Perceived Stress Scale, making it the most frequently used measure. Four (21%) studies used DASS, with one of these also measuring salivary cortisol. Other measures used in the studies included the COVID-19 stress score, prenatal distress questionnaire, Pregnancy Stress Rating Scale-36, Visual Analog Stress Scale, and the Stage of Change in Stress Management, each being used in one study.

#### Intervention Outcomes

Among the studies, significant changes in intervention outcomes were observed in 10 cases, while in 8 studies, stress levels decreased but were not statistically significant (Table 3). In the study that used a game for stress reduction [28], the DASS results did not exhibit a significant difference, whereas the salivary cortisol results did.

**Table 3:** Comparisons and outcomes in the included studies.

Author			Intervention group					Control group				Outcomes
			Provided content		Number of participants			Provided content		Number of participants		
					
**Randomized controlled trials**												
	Balderas-Díaz et al [23]	VivEmbarazo app		15 couples			Routine perinatal care (not mHealth^a^ system)		24 couples		Significant difference in: Maternal stress (PSS^b^) ↓ Baby weight at birth ↑ Gestational age at birth ↑ Preterm ↓ Nonsignificant difference Maternal depression (EPDS^c^)	
	Lee et al [24]	Self-care for Pregnant Women at Work (SPWW) app		60			Application that only had surveys		56		At week 4^d^, significant difference in: Pregnancy stress (PDQ^e^) ↓ Pregnancy hassles ↓ Health practice in pregnancy ↑ Nonsignificant difference in: Work stress Fear of childbirth	
	Sun et al [25]	Spirits Healing app		74			WeChat health consultations to control attention		84		At week 8^d^, significant difference in: Depression (EPDS) ↓ Anxiety symptoms (GAD-7^f^) ↓ Position effect ↑ Nonsignificant difference in: Perceived stress (PSS) Negation effect Sleep-related problems Fatigue Prospective memory Fear of childbirth	
	Smith et al [26]	Calm app		33			Routine perinatal care		27		At day 30^d^, significant difference in: Stress (PSS) ↓ Nonsignificant difference in: Depression (HADS^g^) Anxiety (HADS) Sleep disturbance	
	Krusche et al [27]	Website: Be Mindful Online		22 out of 107 respondents who completed the initial survey			Routine perinatal care		50 out of 78 respondents who completed the initial survey		At day 45^d^, significant difference in: Stress (PSS) ↓ Depression (EPDS) ↓ Pregnancy distress ↓ Nonsignificant difference in: Anxiety (GAD-7)	
	Dennis-Tiwary et al [28]	ABMT app		15			App with placebo mode		14		Significant difference in: Stress (salivary cortisol) ↓ Nonsignificant difference in: Stress (DASS^h^) Anxiety (DASS and HAM-A^i^) Depression (DASS)	
	Kia [29]	Mobile-based health educational intervention		40			A PDF file of the educational content		40		Significant difference in: COVID-19 stress score (CSS-18^j^) ↓	
	Mauriello et al [30]	iPad- Healthy Pregnancy: Step by Step		169			Brochures named March of Dimes on the target behaviors		166		At the third trimesterd, nonsignificant difference in: Stress management Fruit and vegetable consumption	
	Chua et al [31]	Parentbot- a digital health care assistant		59 couples			Routine perinatal care		59 couples		At postpartum months 1 and 3^d^, significant difference in Anxiety (State–Trait Anxiety) ↓ Nonsignificant difference in: Stress (PSS) Depression (EPDS) Support Parent-child bonding	
	Park et al [32]	Mindfulness-based mobile intervention		66			Routine perinatal care, wait-list		67		At 4 weeks^d^, significant difference in: Anxiety (DASS-21) ↓ Emotional well-being ↑ Maternal-fetal attachment ↑ Nonsignificant difference in: Stress (DASS-21) Depression (DASS-21) Postnatal depression (EPDS)	
	Tandon et al [33]	Personalized stress management		49			Routine perinatal care		51		At week 1 and postpartum months 1 and 3^d^, significant difference in: Depression (PROMIS^k^) ↓ Perceived stress (PSS-10) ↓ Behavioral activation ↑ Decentering ↑ Mood regulations Nonsignificant difference in: Anxiety (STAI^l^) Social support	
	Tian et al [34]	WeChat mini-program (mobile digital platform)		80 couples			Routine perinatal care		80 couples		At 2 weeks and postpartum week 6^d^, significant difference in: Maternal perceived stress (PSS-10) ↓ Maternal depression (EPDS) ↓ Paternal depression (EPDS) ↓ Mindfulness (FFMQ^m^) ↑ Infant Neuropsychological Development ↑ Nonsignificant difference in: Anxiety (GAD-7) Symptoms of sleep problems Fatigue	
				
	Porter et al [35]	Expectful mindfulness app		12 out of 21 participants who completed at least one meditation			Routine perinatal care		247		At 28 weeks^d^, significant difference in stress (PSS) ↓	
	Jallo et al [36]	Stress coping intervention app		5 out of 15 participants who completed baseline measure			—^n^		—		Significant change in: Maternal stress (VASS^o^) ↓ Nonsignificant change in: Perceived stress (PSS) Stress coping (CSES^p^)	
	Hashemzahi et al [37]	Telemedicine (WhatsApp Messenger)		50			Routine perinatal care		50		Significant difference in: Perceived stress (PSS) ↓ Corona disease anxiety (CDAS^q^) ↓	
	Tsai et al [38]	Web-based antenatal care system		68			Routine perinatal care: face-to-face individual consulting		67		Significant difference in: Pregnancy-related stress (PSRS-36^r^) ↓ Self-efficacy (GSE^s^) ↑	
	Buultjens et al [39]	Perinatal care, education and support (PECS) intervention		40			Routine perinatal care		21		At 36-38 weeks^d^, significant difference in: Depression (DASS and EPDS) Nonsignificant difference in Stress and anxiety (DASS)	
	Kubo et al [40]	Headspace mindfulness app		20			—		—		Significant change in: Perceived stress (PSS-10) ↓ Depression (PHQ-8^t^) ↓ Sleep disturbance (PSQI^u^) ↓ Mindfulness (FFMQ) ↑	
	Barber and Masters-Awatere [41]	Positively Pregnant (PP) mobile app		42			—		—		Significant change in: Stress (DASS) ↓ Nonsignificant change in: Depression and anxiety (DASS) Depression (APDS)	

^a^mHealth: mobile health

^b^PSS: Perceived Stress Scale.

^c^EPDS: Edinburgh Postnatal Depression Scale.

^d^In the case of repeated measures, the closest measured values at the end of the intervention were compared.

^e^PDQ: prenatal distress questionnaire.

^f^GAD-7: Generalized Anxiety Disorder.

^g^HADS: Hospital Anxiety and Depression Scale.

^h^DASS: Depression Anxiety Stress Scale.

^i^HAM-A: Hamilton Anxiety Scale.

^j^CSS-18: COVID-19 stress score.

^k^PROMIS: Patient-Reported Outcomes Measurement Information System Depression Scale.

^l^STAI: State-Trait Anxiety Inventory Scale.

^m^FFMQ: Five Facet Mindfulness Questionnaire.

^n^Not applicable.

^o^VASS: Visual Analog Stress Scale.

^p^CSES: Coping Self-Efficacy Scale.

^q^CDAS: Corona Disease Anxiety Scale.

^r^PSRS-36: Pregnancy Stress Rating Scale-36.

^s^GSE: General Self-Efficacy Scale.

^t^PHQ: patient health questionnaire.

^u^PSQI: Pittsburgh Sleep Quality Index.

### Results of Syntheses

The meta-analysis results of these studies were as follows.

#### Overall

**Figure 3:**
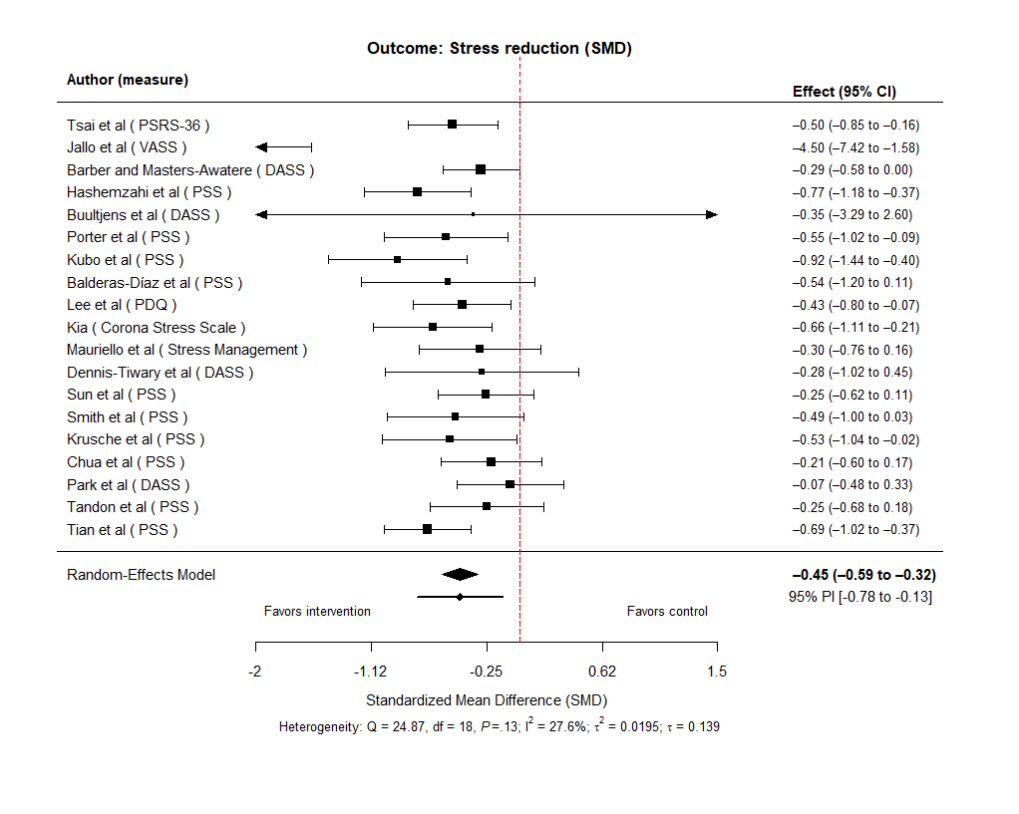
Forest plot of the overall effect of digital interventions on stress reduction [23-41]. DASS: Depression Anxiety Stress Scale; PDQ: prenatal distress questionnaire; PSRS-36: Pregnancy Stress Rating Scale-36; PSS: Perceived Stress Scale; VASS: Visual Analog Stress Scale.

Across 19 studies, effect sizes were calculated and synthesized. A random-effects meta-analysis using the Hartung–Knapp–Sidik–Jonkman method yielded a pooled average effect of SMD –0.45 (95% CI –0.59 to –0.32), indicating a statistically significant reduction in stress (*t*_18_= –6.97, *P*<.001). Between-study heterogeneity was low to moderate (τ²=0.0195, τ=0.1395, *I*²=27.62%, Q (18)=24.87, *P*=.13). The 95% PI ranged from –0.78 to –0.13, indicating that the true effects in new settings are expected to remain beneficial overall, although the magnitude of benefit may vary across implementations (Figure 3 [23-41]).

#### By Strategies

When the intervention effects were categorized by strategies, the pooled estimates were directionally beneficial across subgroups, but statistical certainty varied:

- Education only (k=4): SMD –0.50, 95% CI (–0.73 to –0.27), *I*²=0%, τ²=0
- Education with additional support (k=7): SMD –0.42, 95% CI (–0.84 to 0.00),*I*²=52.8%, τ²=0.059
- Game-based interventions (k=1): SMD –0.28, 95% CI (–1.02 to 0.45),*I*²=0%, τ²=0
- Mindfulness (k=7): SMD –0.48, 95% CI (–0.74 to –0.23),*I*²=39.4%, τ²=0.031

**Figure 4:**
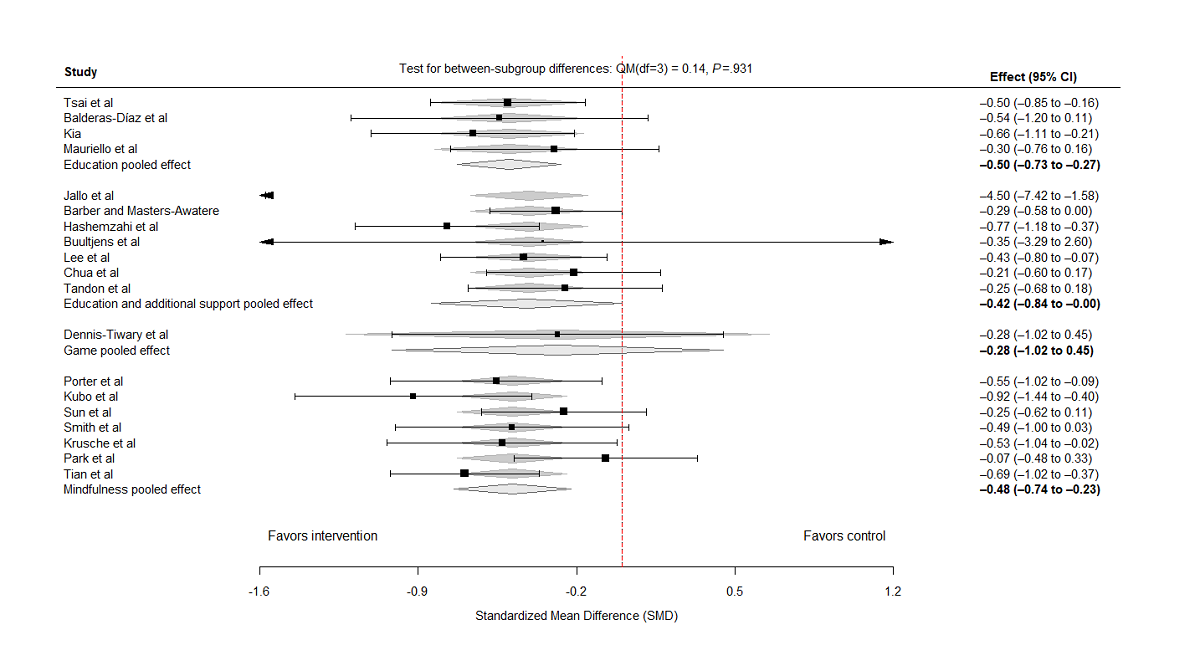
Forest plot for the effects of the intervention by strategies [23-41].

These findings suggest that while several strategies (education-only and mindfulness) show statistically significant average effects, estimates for strategies supported by fewer studies (game-based) or with greater heterogeneity (education with additional support) are less precise and should be interpreted cautiously (Figure 4 [23-41]). A test for between-subgroup differences based on meta-regression showed no statistically significant differences in effect sizes across intervention strategies (Q statistic for moderators: QM_3_=0.14; *P*=.93).

#### Comparison With Routine Antenatal Care

**Figure 5:**
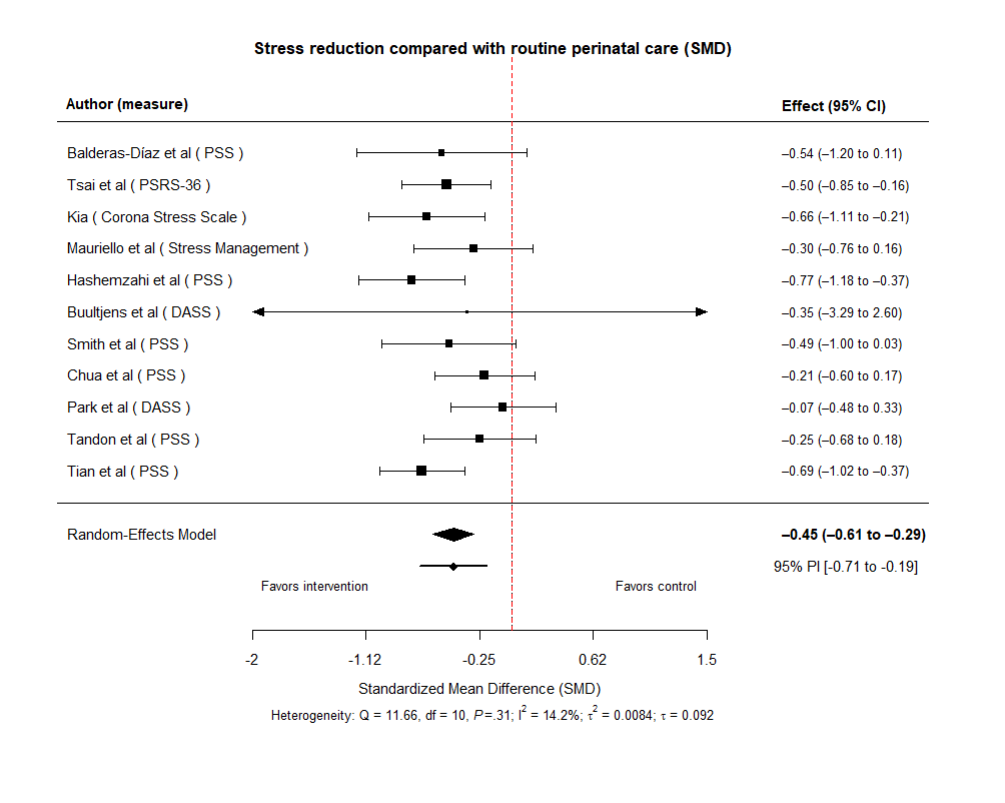
Forest plot for the effects compared with routine perinatal care [23,26,29-34,37-39].

Studies comparing DHIs with routine antenatal care/usual care showed a significant reduction in stress (pooled average effect SMD −0.45, 95% CI −0.61 to −0.29, *t*_10_=−6.27, *P*<.001). Heterogeneity was low (Q (10)=11.66, *P*=.31; *I*²=14.24%, τ²=0.0084; Figure 5 [23,26,29-34,37-39]).

### Certainty of Evidence

Twelve of the 19 included effect sizes were derived from RCTs. Using the GRADE, the certainty of evidence for stress reduction was rated as moderate, downgraded by one level for risk of bias (1/12 RCTs rated overall high risk, and 2/12 rated some concerns in RoB 2), with no serious concerns identified for inconsistency, indirectness, imprecision, or publication bias.

The certainty of evidence was rated as moderate, downgraded by one level for risk of bias. The pooled effect estimate for RCTs was SMD −0.39 (95% CI −0.52 to −0.26), reflecting a moderate level of certainty in the stress-reducing effect of digital interventions (Multimedia Appendix 3).

## Discussion

### Principal Findings

Guided by our objectives to characterize antenatal DHIs for stress management, evaluate their effectiveness, and summarize intervention strategies associated with engagement, this systematic review and meta-analysis found that digitally delivered stress-management interventions are generally effective in reducing stress among pregnant women compared with routine care or control conditions. These findings support the growing role of DHIs as a feasible and scalable approach for addressing pregnancy-related stress. Consistent with an umbrella review of digital interventions for perinatal psychological outcomes, digital approaches appear capable of reducing stress symptoms overall, although prior syntheses often pooled stress with other mental health outcomes [14]. They are partially aligned with a recent systematic review and meta-analysis of digitally delivered mindfulness interventions in pregnant women, which reported clear benefits for depression and anxiety and more variable effects for stress [11]. Our findings extend this evidence by focusing specifically on stress as a primary outcome during pregnancy.

When interventions were classified by dominant strategy, mindfulness-based approaches tended to show the most consistent signals of stress reduction. This pattern aligns with earlier reviews of perinatal mindfulness interventions and with emerging trials of digital mindfulness programs tailored to pregnancy [13,42]. Recent randomized trials suggest that mobile-delivered mindfulness programs can reduce depression, underscoring the potential value of skills-based, self-directed content when engagement is maintained [43]. Education-focused programs were also beneficial, particularly when paired with supportive elements such as coaching, peer interaction, or structured follow-up, suggesting that informational content alone may be insufficient for sustained stress reduction. In contrast, gamified interventions were evaluated in only one study and showed mixed results, with improvements detected in a biological stress indicator but not in self-reported stress, underscoring the need for further research using multiple outcome measures. Importantly, although subgroup-specific point estimates varied across strategies, the formal test for between-subgroup differences was not statistically significant. This indicates insufficient meta-analytic evidence to conclude that any single strategy is superior for stress reduction during pregnancy. Given the limited number of studies within some subgroups and the resulting low statistical power to detect moderator effects, these subgroup patterns should be interpreted cautiously and viewed as exploratory rather than confirmatory.

Beyond intervention strategy, how these programs were delivered also appeared to shape feasibility and engagement. Across studies, most interventions were delivered via mobile apps, with fewer web-based platforms and telemedicine approaches, reflecting contemporary trends in digital health delivery for pregnant populations. The predominance of mobile app–based interventions observed in this review mirrors widespread smartphone use among pregnant women and supports the feasibility of delivering brief, scalable stress-management content within routine antenatal care [16,44]. The increasing dominance of mobile-delivered interventions in more recent studies highlights a shift toward app-based platforms that can leverage a wider range of interactive functions—such as continuous monitoring, automated feedback, and reminders—which may enhance engagement and responsiveness compared with more static web-based programs. While earlier meta-analyses of internet-delivered psychological interventions primarily emphasized benefits for depression and anxiety outcomes [45], our findings suggest that web-based interventions can also contribute to stress reduction during pregnancy, albeit based on a smaller number of trials.

Beyond delivery mode alone, consistent patterns emerged in how interventions were constructed. Most DHIs combined mobile or web-based delivery with self-guided use, supported by functional features such as self-monitoring, automated feedback, reminders, or asynchronous communication, and delivered therapeutic content including psychoeducation, mindfulness practices, relaxation techniques, or cognitive behavioral therapy–based skills. Multi-component designs were the norm rather than the exception, indicating that digital stress-management interventions during pregnancy rarely rely on a single active ingredient. Compared with earlier reviews that grouped interventions primarily by delivery methods or broad perinatal mental health outcomes [13,46], the present review adopts a strategy-oriented perspective that provides a more clinically meaningful framework for understanding how digital interventions may be designed to reduce stress during pregnancy. These findings highlight the need for future research that directly compares single-strategy interventions (such as mindfulness alone or psychoeducation alone) with multi-component interventions, in order to clarify whether additive or synergistic effects contribute to stress reduction in this population.

Interpretation of these findings should consider both between-study variability and study quality. Although between-study variability did not indicate extreme inconsistency, the prediction interval suggests that beneficial effects are likely across future settings, albeit with meaningful variation in magnitude. Reporting prediction intervals alongside CIs helps distinguish the average effect from the distribution of effects expected in real-world implementations [19]. Subgroup findings should therefore be interpreted in the context of overall consistency rather than as definitive evidence of differential effectiveness across strategies [47]. Finally, assessments of small-study effects should be interpreted cautiously, as funnel plot asymmetry may reflect mechanisms beyond publication bias, including true heterogeneity or methodological differences across studies [48].

### Clinical Implications

These results suggest that DHIs can serve as a practical adjunct to routine antenatal care by extending access to evidence-based stress-management skills, particularly for women who face barriers to in-person services. Interventions that combine structured mindfulness practice or targeted education with functional support (eg, reminders, progress feedback, brief coaching, or moderated peer support) may be especially useful for maintaining adherence and reinforcing skills [16]. Implementation may be facilitated by integrating these tools into prenatal education pathways and ensuring appropriate guidance for women with elevated distress who may need stepped-up care. Recent trials suggest that mobile-delivered mindfulness may improve depressive symptoms during pregnancy, but sustained engagement and equitable access remain key implementation challenges [43]. From a design perspective, the most promising programs provided clear goals, brief and repeatable programs, and opportunities for personalization. Future DHIs could also explore safe integration of passive sensing or wearable-enabled feedback to tailor content in real time, while ensuring data privacy and minimizing user burden [47,48]. Recent trials also highlight the practical importance of reporting engagement metrics (eg, module completion, frequency of practice, and prompt response) so that implementation decisions can be based not only on efficacy but also on real-world use patterns [43].

### Limitations

Several limitations should be noted. The number of trials within some strategy subgroups was small, limiting precision and the ability to detect differential effects. Our restriction to English-language publications and the use of only 4 databases may have missed relevant studies, and most included trials were conducted in high-income countries, which may limit generalizability to low- and middle-income contexts. Outcomes were primarily self-reported, raising the possibility of reporting bias. In addition, incomplete reporting of intervention details and adherence in some trials constrained the interpretation of which components drove benefit. Because the review was not prospectively registered, transparency may be lower than in preregistered reviews; however, we sought to mitigate this limitation by adhering to PRISMA guidance and applying the GRADE approach to characterize certainty of evidence. Moreover, given the rapid evolution of digital perinatal interventions, studies published after our search window may influence future pooled estimates; ongoing evidence surveillance or living-review approaches may therefore be warranted [14,43].

### Conclusions

Overall, this systematic review and meta-analysis provide novel evidence that digitally delivered stress-management interventions can meaningfully reduce stress during pregnancy, highlighting the potential of digital health approaches as a complementary component of antenatal care. Unlike previous reviews that primarily categorized interventions by delivery platform or aggregated stress with other psychological outcomes, this study offers an innovative strategy-based synthesis that disentangles how specific intervention contents and supportive features contribute to stress reduction in pregnant women. By distinguishing intervention strategies rather than technologies alone, the findings advance understanding of what works within digital stress-management programs during pregnancy.

Across studies, the most consistent benefits were observed for mindfulness-based approaches and for educational interventions combined with supportive features such as coaching or peer interaction, underscoring the importance of active skill-building and engagement rather than information provision alone. Importantly, while digital tools are not intended to replace clinician-delivered care, the results indicate that they can extend access to evidence-based coping strategies and provide scalable, low-intensity support that fits within real-world antenatal workflows. As mobile app–based interventions continue to expand, their ability to integrate multiple functions—such as self-monitoring, feedback, and reminders—positions them as particularly feasible and adaptable tools for routine maternity care.

By clarifying the effectiveness of digital stress-management interventions specifically during pregnancy and by framing interventions according to their strategic components, this review contributes actionable insights for clinicians, researchers, and health system planners. The findings support the integration of digital stress-management programs as an adjunct to standard antenatal services, with the potential to improve reach, equity, and continuity of psychosocial support for pregnant women in diverse care settings.

## Data Availability

The data that support the findings of this study are available from the corresponding author upon reasonable request.

## Appendix

Multimedia Appendix 1 PRISMA 2020 Checklist and PRISMA-S Checklist.

Multimedia Appendix 2 Detailed Search Strategies across Databases.

Multimedia Appendix 3 Effect Estimates from Randomized Controlled Trials with GRADE Quality Assessment.

## Abbreviations

DASS: Depression Anxiety Stress Scale
DHI: digital health intervention
GRADE: Grading of Recommendations, Assessment, Development and Evaluation
MeSH: Medical Subject Headings
PICOS: population, intervention, comparison, outcome, and study design
PRISMA: Preferred Reporting Items for Systematic Reviews and Meta-Analyses
PRISMA-S: Preferred Reporting Items for Systematic Reviews and Meta-Analyses literature search extension
RoB 2: revised Cochrane risk-of-bias tool
ROBINS-I: risk of bias in nonrandomized studies of interventions
SMD: standardized mean difference
