# Supplementary material for: Evaluating the Effectiveness of Digital Interventions for Stress Management in Pregnant Women: Systematic Review and Meta-Analysis
Source: JMIR Mhealth Uhealth. 2026 Jan 26;14:e66267. doi: 10.2196/66267 (PMC12887566; doi:10.2196/66267)
Supplement: Multimedia Appendix 2 [file mhealth_v14i1e66267_app2.docx]

| **Pubmed** |
| --- |
| ((("perinatal care"[MeSH Terms] OR "perinatal care*"[Title/Abstract] OR ("prenatal care"[MeSH Terms] OR "prenatal care*"[Title/Abstract]) OR ("pregnancy"[MeSH Terms] OR ("pregnanc*"[Title/Abstract] OR "gestation*"[Title/Abstract])) OR ("pregnant women"[MeSH Terms] OR "pregnant wom*"[Title/Abstract]) OR "expectant mother*"[Title/Abstract] OR "perinatal wom*"[Title/Abstract] OR "conceive*"[Title/Abstract]) AND ("computers, handheld"[MeSH Terms] OR ("palm pilot*"[Title/Abstract] OR "palm top computer*"[Title/Abstract] OR "pda computer*"[Title/Abstract] OR "pocket pc*"[Title/Abstract] OR "digital device*"[Title/Abstract] OR "handheld computer*"[Title/Abstract] OR "personal digital assistant*"[Title/Abstract] OR "palmtop computer*"[Title/Abstract] OR "tablet computer*"[Title/Abstract]) OR "portable computer*"[Title/Abstract] OR ("mobile applications"[MeSH Terms] OR "mobile app*"[Title/Abstract]) OR ("smartphone"[MeSH Terms] OR "smartphone"[Title/Abstract]) OR ("telemedicine"[MeSH Terms] OR ("telemedicine*"[Title/Abstract] OR "mhealth*"[Title/Abstract] OR "mobile health*"[Title/Abstract] OR "ehealth*"[Title/Abstract] OR "telehealth*"[Title/Abstract])) OR "e health*"[Title/Abstract] OR "digital health*"[Title/Abstract] OR "digital intervention*"[Title/Abstract] OR "electronic health*"[Title/Abstract] OR "healthcare software*"[Title/Abstract] OR "electronic app*"[Title/Abstract] OR "handheld instrument*"[Title/Abstract] OR "m health*"[Title/Abstract] OR "mobile intervention*"[Title/Abstract]) AND ("stress"[Title/Abstract] OR ("stress, psychological"[MeSH Terms] OR "psychological stress*"[Title/Abstract]) OR ("psychotherapy"[MeSH Terms] OR "psychotherap*"[Title/Abstract]) OR ("mental health"[MeSH Terms] OR "mental health*"[Title/Abstract]) OR "strain*"[Title/Abstract])) NOT ("animals"[MeSH Terms] NOT "humans"[MeSH Terms])) AND (english[Filter]) |
| **Embase** |
| ((('perinatal care'/exp OR 'perinatal care*':ab,ti) OR ('prenatal care'/exp OR 'prenatal care*':ab,ti) OR ('pregnancy'/exp OR (pregnanc*:ab,ti OR gestation*:ab,ti)) OR ('pregnant woman'/exp OR 'pregnant wom*':ab,ti) OR ('expectant mother'/exp OR 'expectant mother*':ab,ti) OR 'perinatal wom*':ab,ti OR conceive*:ab,ti) AND ('palm pilot*':ab,ti OR 'palm top computer*':ab,ti OR 'pda computer*':ab,ti OR 'pocket pc*':ab,ti OR ('digital device'/exp OR 'digital device*':ab,ti) OR ('personal digital assistant'/exp OR ('handheld computer*':ab,ti OR 'personal digital assistant*':ab,ti)) OR 'palmtop computer*':ab,ti OR ('tablet computer'/exp OR 'tablet computer*':ab,ti) OR 'portable computer*':ab,ti OR ('mobile application'/exp OR 'mobile app*':ab,ti) OR ('smartphone'/exp OR smartphone:ab,ti) OR ('telemedicine'/exp OR telemedicine*:ab,ti) OR ('mhealth'/exp OR mhealth*:ab,ti) OR ('mobile health'/exp OR 'mobile health*':ab,ti) OR ('telehealth'/exp OR (ehealth*:ab,ti OR telehealth*:ab,ti OR 'e health*':ab,ti)) OR ('digital health'/exp OR 'digital health*':ab,ti) OR ('digital intervention'/exp OR 'digital intervention*':ab,ti) OR ('electronic health'/exp OR 'electronic health*':ab,ti) OR ('healthcare software'/exp OR 'healthcare software*':ab,ti) OR 'electronic app*':ab,ti OR 'handheld instrument*':ab,ti OR 'm health*':ab,ti OR 'mobile intervention*':ab,ti) AND (('physiological stress'/exp OR (stress:ab,ti OR 'psychological stress*':ab,ti)) OR ('psychotherapy'/exp OR psychotherap*:ab,ti) OR ('mental health'/exp OR 'mental health*':ab,ti) OR ('strain'/exp OR strain*:ab,ti))) NOT ('animal'/exp NOT 'human'/exp) AND [english]/lim |
| **Cochrane** |
| ((((([mh "Perinatal Care"] OR (perinatal next care*):ab,ti) OR ([mh "Prenatal Care"] OR (prenatal next care*):ab,ti) OR ([mh Pregnancy] OR (pregnanc*:ab,ti OR gestation*:ab,ti)) OR ([mh "Pregnant Women"] OR (pregnant next wom*):ab,ti) OR (expectant next mother*):ab,ti OR (perinatal next wom*):ab,ti OR conceive*:ab,ti)) AND ((([mh "Computers, Handheld"] OR ((palm next pilot*):ab,ti OR (palm next top next computer*):ab,ti OR (PDA next computer*):ab,ti OR (pocket next PC*):ab,ti OR (digital next device*):ab,ti OR (handheld next computer*):ab,ti OR (personal next digital next assistant*):ab,ti OR (palmtop next computer*):ab,ti OR (tablet next computer*):ab,ti)) OR (portable next computer*):ab,ti OR ([mh "Mobile Applications"] OR (mobile next app*):ab,ti) OR ([mh Smartphone] OR smartphone:ab,ti) OR ([mh Telemedicine] OR (telemedicine*:ab,ti OR mhealth*:ab,ti OR (mobile next health*):ab,ti OR ehealth*:ab,ti OR telehealth*:ab,ti)) OR (e next health*):ab,ti OR (digital next health*):ab,ti OR (digital next intervention*):ab,ti OR (electronic next health*):ab,ti OR (healthcare next software*):ab,ti OR (electronic next app*):ab,ti OR (handheld next instrument*):ab,ti OR (m next health*):ab,ti OR (mobile next intervention*):ab,ti)) AND ((stress:ab,ti OR ([mh "Stress, Psychological"] OR (psychological next stress*):ab,ti) OR ([mh Psychotherapy] OR psychotherap*:ab,ti) OR ([mh "Mental Health"] OR (mental next health*):ab,ti) OR strain*:ab,ti))) NOT (([mh Animals] NOT [mh Humans]))) |
| **CINAHL** |
| "(MH ""Perinatal Care"" OR TI ""perinatal care*"" OR AB ""perinatal care*"" OR MH ""Prenatal Care"" OR TI ""prenatal care*"" OR AB ""prenatal care*"" OR MH ""Pregnancy"" OR TI pregnanc* OR AB pregnanc* OR TI gestation* OR AB gestation* OR MH ""Expectant Mothers"" OR TI ""pregnant wom*"" OR AB ""pregnant wom*"" OR TI ""expectant mother*"" OR AB ""expectant mother*"" OR TI ""perinatal wom*"" OR AB ""perinatal wom*"" OR TI conceive* OR AB conceive*)  AND  (TI ""palm pilot*"" OR AB ""palm pilot*"" OR TI ""palm top computer*"" OR AB ""palm top computer*"" OR TI ""PDA computer*"" OR AB ""PDA computer*"" OR TI ""pocket PC*"" OR AB ""pocket PC*"" OR TI ""digital device*"" OR AB ""digital device*"" OR MH ""Computers, Hand-Held"" OR TI ""handheld computer*"" OR AB ""handheld computer*"" OR TI ""personal digital assistant*"" OR AB ""personal digital assistant*"" OR TI ""palmtop computer*"" OR AB ""palmtop computer*"" OR MH ""Computers, Portable"" OR TI ""tablet computer*"" OR AB ""tablet computer*"" OR TI ""portable computer*"" OR AB ""portable computer*"" OR MH ""Mobile Applications"" OR TI ""mobile app*"" OR AB ""mobile app*"" OR MH ""Smartphone"" OR TI smartphone OR AB smartphone OR MH ""Telemedicine"" OR TI telemedicine* OR AB telemedicine* OR MH ""Telehealth"" OR TI mhealth* OR AB mhealth* OR TI ""mobile health*"" OR AB ""mobile health*"" OR TI ehealth* OR AB ehealth* OR TI telehealth* OR AB telehealth* OR TI ""e health*"" OR AB ""e health*"" OR MH ""Digital Health"" OR TI ""digital health*"" OR AB ""digital health*"" OR TI ""digital intervention*"" OR AB ""digital intervention*"" OR TI ""electronic health*"" OR AB ""electronic health*"" OR TI ""healthcare software*"" OR AB ""healthcare software*"" OR TI ""electronic app*"" OR AB ""electronic app*"" OR TI ""handheld instrument*"" OR AB ""handheld instrument*"" OR TI ""m health*"" OR AB ""m health*"" OR TI ""mobile intervention*"" OR AB ""mobile intervention*"")  AND  (MH ""Stress"" OR TI stress OR AB stress OR MH ""Stress, Psychological"" OR TI ""psychological stress*"" OR AB ""psychological stress*"" OR MH ""Psychotherapy"" OR TI psychotherap* OR AB psychotherap* OR MH ""Mental Health"" OR TI ""mental health*"" OR AB ""mental health*"" OR TI strain* OR AB strain*)  AND NOT  (MH ""Animals"" AND NOT MH ""Human"")" |
