# Supplementary material for: Evaluating the Effectiveness of Digital Interventions for Stress Management in Pregnant Women: Systematic Review and Meta-Analysis
Source: JMIR Mhealth Uhealth. 2026 Jan 26;14:e66267. doi: 10.2196/66267 (PMC12887566; doi:10.2196/66267)

| **Certainty assessment** | | | | | | | **№ of patients** | | **Effect** | **Certainty** | **Importance** |
| --- | --- | --- | --- | --- | --- | --- | --- | --- | --- | --- | --- |
| **No. of Studies** | **Study design** | **Risk of Bias** | **Inconsistency** | **Indirectness** | **Imprecision** | **Publication Bias** | **No. of Participants**  **(Intervention)** | **No. of Participants**  **(Control)** | **SMD**  **(95% CI)** |  |  |
| 12 | Randomized trials | Serious* | Not serious† | Not serious | Not serious† | Undetected | 520 | 535 | -0.39  [ -0.52, -0.26 ] | Moderate | Critical |

CI, confidence interval; GRADE, Grading of Recommendations Assessment, Development and Evaluation; NA, not applicable; RR, relative risk; SMD, standardized mean difference.

* Downgraded one level for risk of bias: 1/12 studies rated overall high risk (randomization domain) and 2/12 rated some concerns across multiple domains in RoB 2.


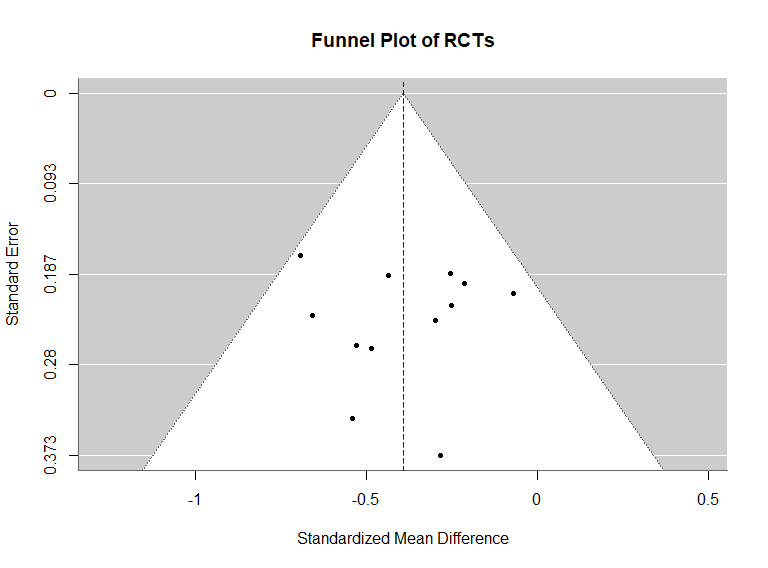

Supplement: Multimedia Appendix 3 [file mhealth_v14i1e66267_app3.docx]
